# XIST Inhibition Attenuates Calcium Oxalate Nephrocalcinosis-Induced Renal Inflammation and Oxidative Injury via the miR-223/NLRP3 Pathway

**DOI:** 10.1155/2021/1676152

**Published:** 2021-09-02

**Authors:** Peng Lv, Haoran Liu, Tao Ye, Xiaoqi Yang, Chen Duan, Xiangyang Yao, Bo Li, Kun Tang, Zhiqiang Chen, Jianhe Liu, Yaoliang Deng, Tao Wang, Jinchun Xing, Chaozhao Liang, Hua Xu, Zhangqun Ye

**Affiliations:** ^1^Department of Urology, Tongji Hospital, Tongji Medical College, Huazhong University of Science and Technology, Wuhan, China; ^2^Department of Urology, The First Affiliated Hospital of Anhui Medical University, Institute of Urology, Anhui Medical University, Hefei, China; ^3^Department of Urology, The Second Affiliated Hospital of Kunming Medical University, Kunming, China; ^4^Department of Urology, The First Affiliated Hospital of Guangxi Medical University, Nanning, China; ^5^Department of Urology, The First Affiliated Hospital of Xiamen University, Xiamen, China

## Abstract

The roles of the lncRNA X inactive specific transcript (XIST) in many diseases, including cancers and inflammatory sickness, have been previously elucidated. However, renal calculus remained poorly understood. In this study, we revealed the potential effects of XIST on kidney stones that were exerted via inflammatory response and oxidative stress mechanisms. We established a glyoxylate-induced calcium oxalate (CaOx) stone mouse model and exposed HK-2 cells to calcium oxalate monohydrate (COM). The interactions among XIST, miR-223-3p, and NOD-like receptor protein 3 (NLRP3) and their respective effects were determined by RNAs and protein expression, luciferase activity, and immunohistochemistry (IHC) assays. Cell necrosis, reactive oxygen species (ROS) generation, and inflammatory responses were detected after silencing XIST, activating and inhibiting miR-223-3p, and both knocking down XIST and activating miR-223-3p in vitro and in vivo. The XIST, NLRP3, caspase-1, and IL-1*β* levels were notably increased in kidney samples from glyoxylate-induced CaOx stone model mice. XIST knockdown significantly suppressed the inflammatory damage and ROS production and further attenuated oxalate crystal deposition. miRNA-223-3p mimics also exerted the same effects. Moreover, we verified the interactions among XIST, miRNA-223-3p and NLRP3, and the subsequent effects. Our results suggest that the lncRNA XIST participates in the formation and progression of renal calculus by interacting with miR-223-3p and the NLRP3/Caspase-1/IL-1*β* pathway to mediate the inflammatory response and ROS production.

## 1. Introduction

The prevalence of kidney stones disease among adults in China has been approximately 10.63% in recent decades, and this prevalence continues to increase; the recurrence rate in the first 5 years after the first kidney stone is also continuously increasing [1, 2]. Calcium oxalate (CaOx) stones, which account for 80% of all renal calculus cases, induce a renal inflammatory response, resulting in renal tubular cell necrosis and additional CaOx crystal deposition [3, 4]. The NLRP3 protein was first identified in autoimmune diseases and recruited the adaptor molecule ASC through PYD-PYD interactions, then recruited procaspase-1 to form the inflammasome complex [5, 6]. Activated caspase-1 participates in the processing of the cytokine prointerleukin-1*β* into its mature and active secreted form [7]. Increasing evidence suggests that NLRP3 plays a significant role in the pathogenesis of autoinflammatory, autoimmune, and chronic inflammatory and metabolic diseases [8–10].

Competing endogenous RNAs (ceRNAs) may play critical roles in RNA-RNA interactions and could explain the relationship among long noncoding RNAs (lncRNAs), miRNAs, and mRNAs [11]. The 3′-untranslated regions (UTRs) of lncRNAs and mRNAs have common base sites at which miRNAs can bind and thereby influence the expression of downstream proteins [12, 13]. The lncRNA XIST is indispensable for X-chromosome inactivation in mammals, which contributes to regulating the associated RNA-binding proteins (RBPs), chromatin modification, and gene silencing [14, 15]. Emerging evidence suggests that XIST participates in regulating inflammation, tissue injury, and cancer [16–18]. Moreover, Shen et al. reported that silencing XIST attenuated sepsis-induced acute liver injury and prevented inflammatory responses, ROS production, and cell apoptosis by inhibiting the BRD4 expression [19]. Nevertheless, the mechanism by which XIST regulates CaOx-induced renal inflammation and injury is largely unknown.

In this research, we studied the competing interactions among XIST/miRNA-223/NLRP3 in CaOx monohydrate-treated HK-2 cells and a glyoxylate-induced CaOx mouse model. We found that XIST sponges miR-223-3p to enhance renal tubular epithelial cell inflammatory responses and damage by activating the NLRP3/Caspase-1/IL-1*β* pathway.

## 2. Materials and Methods

### 2.1. Cell Culture and Transfection

HK-2 cells were purchased from American Type Culture Collection (ATCC, USA) and maintained in Roswell Park Memorial Institute 1640 medium (HyClone, UT) supplemented with 10% fetal bovine serum (Sigma, USA) at 37°C in a humid 5% CO_2_ atmosphere. Cell viability was assessed with a Cell Counting Kit (Beyotime, China). To silence the expression of XIST, a validated small interfering RNA (siRNA) specifically targeting XIST (si-XIST, GCACAAACTCAATTCTCTA) was synthesized by RiboBio (Guangzhou, China). To overexpress or knock down miRNA-223, synthetic miRNA oligonucleotides (miR-223-3p mimic: 5′-3′: UGUCAGUUUGUCAAAUACCCCA, miR-223-3p inhibitor: 5′-3′: UGUCAGUUUGUCAAAUACCCCA) and the corresponding negative controls were purchased from RiboBio (Guangzhou, China). We transfected chemosynthetic siRNA or miRNA oligonucleotides by using Lipofectamine 3000 (Invitrogen, USA).

### 2.2. Animal Experiments

Our experiments were conducted in accordance with the criteria established by the National Institutes of Health Guide for the Care and Use of Laboratory Animals. Then, eight-week-old C57BL/6J male mice were raised under specific pathogen-free conditions at the Laboratory Animal Center of Tongji Hospital. Each mouse received saline or glyoxylate via intraperitoneal injection every day for one week. Eight-week-old mice in the treatment group were intravenously injected with 3 × 10^12^ viral genomes of rAAV-sh-XIST or a rAAV vector as a negative control (produced by Vigene, China) in a volume of 100 *μ*l via the tail vein. Two weeks after the rAAV injection, immunofluorescence analysis was performed to confirm that the mouse kidneys were infected with rAAV (Supplementary Figure S1). In the miRNA-223 intervention group, a long-acting miRNA-223 antagonist was injected via the tail vein on days 1 and 4. The mice were sacrificed and dissected after one week, and bilateral kidney specimens were then fixed in 4% paraformaldehyde for further experiments. The animal study was approved by the Ethics Committee of Tongji Hospital.

### 2.3. CaOx Crystal Measurement

Renal paraffin sections were dewaxed and stained with hematoxylin-eosin according to the standard experimental procedure. The stained samples were then observed under a polarizing microscope (Zeiss, Germany). Moreover, stained renal paraffin sections were analyzed by the Pizzolato method to verify the amount of CaOx crystal deposition [20]. Finally, the quantity of CaOx crystals was determined with ImageJ software (National Institutes of Health, USA).

### 2.4. Tubular Injury and Necrosis Detection

The renal paraffin sections were dewaxed and then subjected to periodic acid-Schiff (PAS) staining. Kidney damage was scored according to the following four aspects: tubular necrosis, epithelial cell apoptosis, intraluminal cast formation, and brush border loss. We randomly selected nonoverlapping microscopic fields of view and calculated the ratio of tubular injury. Grades 0 to 5 represented 0%, ≤10%, 11–25%, 26–45%, 46–75%, and ≥76% injury, respectively. Cell necrosis in the renal section samples was assessed via TdT-mediated dUTP nick-end labeling (TUNEL). Positive cells were counted in ten randomly selected nonoverlapping microscopic fields of view.

### 2.5. Real-Time Quantitative PCR

Total RNA was collected by using TRIzol Reagent (Invitrogen, USA) according to the standard experimental procedure, and complementary DNA was synthesized by using an All-in-one RT SuperMix Perfect RT-PCR Kit (Vazyme, China). Real-time quantitative PCR was performed with SYBR Green Master Mix (Yeasen, 11201-11203) to assess RNA expression levels on a Bio-Rad CFX96 system, and the target RNA levels were normalized to those of GAPDH or U6. The sequences of all the primers are listed in Supplementary Table 1.

### 2.6. Flow Cytometry

HK-2 cells were cultured in six-well plates together with 100 *μ*g/ml COM (Sigma-Aldrich, USA) and treated with various transfection reagents for 2 h. Next, the cells were treated with 2,7-dichlorofluorescein diacetate (DCFH-DA, BD Bioscience, USA) for 30 minutes at 37°C. Finally, internal ROS production in the cells was detected via flow cytometry at wavelengths of 485 nm and 538 nm. HK-2 cells were cultured in six-well plates together with 100 *μ*g/ml COM for 24 h and then stained with an Annexin V-FITC and PI Apoptosis Detection Kit (BD, USA). The necrosis of entire whole population of stained cells was monitored by flow cytometry (BD, USA).

### 2.7. Determination of Superoxide Dismutase (SOD), Lactate Dehydrogenase (LDH), Malondialdehyde (MDA), and Hydrogen Peroxide (H_2_O_2_) Levels

The levels of LDH and MDA, which are used to assess cell membrane destruction and lipid peroxidation, respectively, in the supernatants of cultured HK-2 cells were measured using commercial LDH and MDA assay kits (Beyotime Biotech, China) according to the manufacturer's instructions. To further measure the antioxidative enzyme activities in HK-2 cells, the SOD and H_2_O_2_ levels were assessed using commercially available assay kits (Beyotime Biotech, China) according to the respective manufacturer's instructions.

### 2.8. Measurement of ROS Generation

Treated HK-2 cells were cultured with DCFH-DA (S0033, Beyotime Biotech, China) for 30 minutes. Intracellular ROS generation in tubular epithelial cells (TECs) was detected by flow cytometry (BD Bioscience, USA) with excitation and emission wavelengths of at 488 and 525 nm, respectively. Intracellular ROS generation was also monitored by using a fluorescence microscope (Nikon, Japan). The levels of ROS generation were quantified with ImageJ software.

### 2.9. Mitochondrial Membrane Potential Assay

Tetramethyl rhodamine ethyl ester (TMRE) (ab113852, Abcam, USA) was used to determine the mitochondrial membrane potential. In brief, treated TECs were incubated with 150 nM TMRE diluted in warm culture medium for 15 minutes. The cells were then washed twice with warm PBS, and the fluorescence intensity was detected using a plate reader at excitation/emission wavelengths of 520/590 nm.

### 2.10. Western Blot Analysis

Whole protein was harvested from HK-2 cells with RIPA buffer containing a complete protease inhibitor cocktail (Servicebio, China), and the protein concentrations were determined by using a BCA Kit (Beyotime, China). The protein samples were separated and then incubated with antibodies against NLRP3 (Boster, China, BM4490), caspase-1 (Absin, China, abs119750), IL-1*β* (Absin, China), and GAPDH (Absin, China, abs132004) at 4°C overnight. Next, the cells were incubated with the appropriate corresponding horseradish peroxidase-conjugated secondary antibodies (Cell Signaling Technology, USA) at room temperature for 2 h and visualized with an ECL solution (Millipore, USA) on a ChemiDoc XRS instrument (Bio-Rad, USA). The relative expression levels of the proteins were analyzed with ImageJ software (National Institutes of Health, USA).

### 2.11. Dual-Luciferase Reporter Gene Assay

To construct the luciferase reporter vectors, the 3′-UTRs of XIST and NLRP3, which included a wild-type putative miR-223-3p binding site and an artificially mutated putative binding site, were inserted into the pGL4.13 plasmid. Then, HK-2 cells were cotransfected with the plasmids and artificial miR-223-3p mimics and inhibitor. After 2 days of transfection, the Dual-Luciferase Reporter Assay System (Promega, Madison, WI, USA) was used to detect the luciferase activity. The firefly luciferase activity is presented as the relative expression level and was normalized to that of the Renilla luciferase activity.

### 2.12. Immunohistochemistry

Renal paraffin sections were dewaxed and processed according to the standard experimental procedure, and the samples were incubated with the anti-NLRP3 (1 : 400, Servicebio, China, GB11300), anti-caspase-1 (1 : 1000, Servicebio, China, GB11383), anti-IL-1*β* (1 : 600, Servicebio, China, GB11113), anti-SOD2 (1 : 200, Boster, China, BM4813), and anti-NOX2 (1 : 600, Boster, China, BA2811) antibodies overnight at 4°C. The relative expression of the corresponding proteins was analyzed with ImageJ software (National Institutes of Health, USA).

### 2.13. RNA Fluorescence In Situ Hybridization (FISH)

After the injection of mice with different doses of COM, kidney samples were harvested and fixed in paraffin wax. The sections were dewaxed and then treated with protease K at 37°C for 20 minutes. Denaturation was performed with denatured solutions containing different percentages of ethyl alcohol at -20°C. The sections were incubated with probes at 37°C for 12 h and then washed three times. Fluorescent labeling and nuclear staining were used to observe the differences in RNA expression among the groups.

## 3. Statistical Analysis

GraphPad Prism 8.4 and Stata SE15.1 were used to analyze the data. The experimental data are shown as the mean ± SD. We performed *t*-tests and one-way analysis of variance (ANOVA) to compare the differences among diverse groups. *P* values <0.05 were considered as statistically significant.

## 4. Results

### 4.1. XIST and NLRP3 Expression Was Significantly Increased in the CaOx Nephrocalcinosis Mouse Model

**Figure 1:**
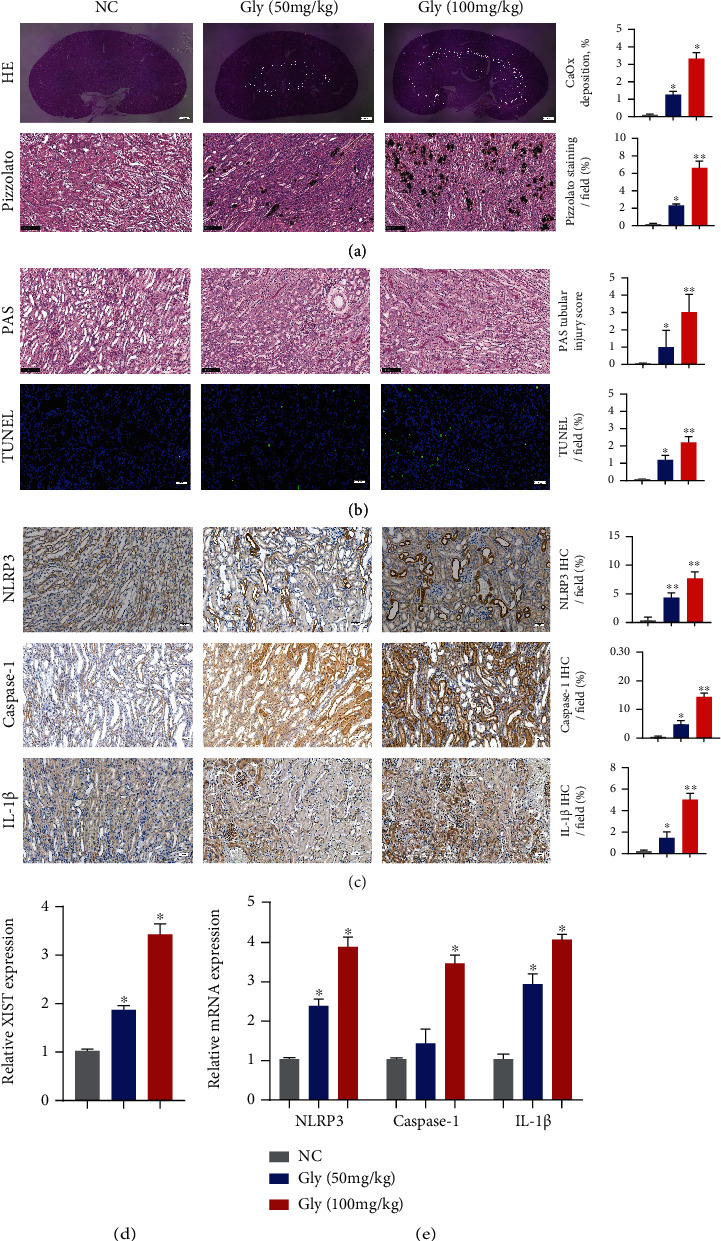
XIST and NLRP3 expression was significantly increased in the CaOx nephrocalcinosis mouse model. (a) Polarizing microscopy and Pizzolato staining were performed to verify the crystal deposition after the administration of glyoxylate at varying concentrations to CaOx nephrocalcinosis model mice. (b) PAS and TUNEL staining was used to observe the kidney injury. (c) The levels of NLRP3, Caspase-1, and IL-1*β* and the positive ratio were detected by IHC. (d, e) The expression levels of XIST, NLRP3, Caspase-1, and IL-1*β* in kidney samples were determined by qPCR. The data are presented as the mean ± SD of three independent experiments. ^∗^*P* < 0.05; ^∗∗^*P* < 0.01, as assessed via Student's *t*-test (a–c) and one-way ANOVA (d, e).

We established a glyoxylate-induced CaOx nephrocalcinosis mouse model by injecting mice with different doses of glyoxylate (0 mg/kg, 50 mg/kg, and 100 mg/kg) for seven days. Then, we performed HE staining and Pizzolato staining and then observed under a polarized optical microscope that the quantity of CaOx increased with increasing doses of glyoxylate (Figure 1(a)). Additionally, PAS staining showed that the injury caused by CaOx became more extensive after glyoxylate injection (Figure 1(b)). TUNEL staining revealed significant cell death after glyoxylate injection (Figure 1(b)). Moreover, immunohistochemistry analysis revealed high expression levels of NLRP3, caspase-1, and IL-1*β* in nephrocalcinosis samples (Figure 1(c)). We also found that the RNA expression levels of XIST, NLRP3, caspase-1, and IL-1*β* were highly elevated in the samples of glyoxylate-induced CaOx kidney stones (Figures 1(d) and 1(e)).

### 4.2. Inhibition of XIST Attenuated CaOx-Induced Renal Inflammation and Oxidative Injury In Vitro

**Figure 2:**
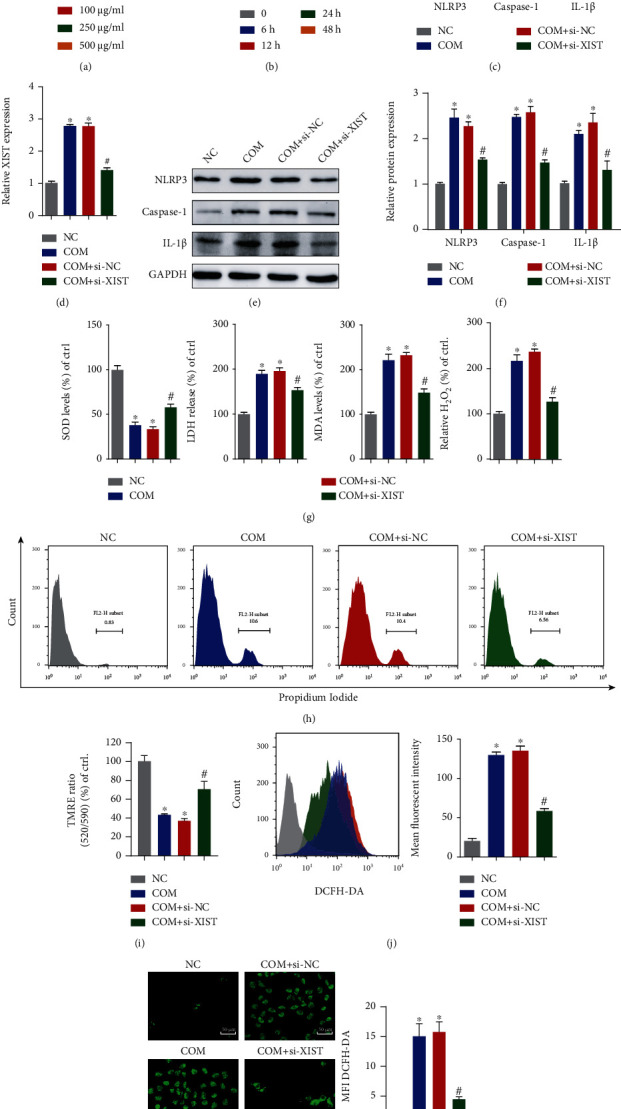
Inhibition of XIST attenuated CaOx-induced renal inflammation and oxidative injury in vitro. COM at various doses (0 *μ*g/ml, 50 *μ*g/ml, 100 *μ*g/ml, 250 *μ*g/ml, and 500 *μ*g/ml) was cocultured with HK-2 cells, and the cell viability was detected. (b) Cells were cocultured with 100 *μ*g/ml COM for different amounts of time (0 h, 6 h, 12 h, 24 h, and 48 h), and the cell viability was examined. (c, d) qRT-PCR was used to detect the expression of XIST, NLRP3, Caspase-1, and IL-1*β* in HK-2 cells. (e, f) Western blotting also showed changes in the protein levels of NLRP3, Caspase-1, and IL-1*β* in HK-2 cells. (g) The generation of SOD, LDH, MDA, and H_2_O_2_ after different treatments was examined. (h) Flow cytometry was performed to assess cell necrosis. (i) TMRE was used to determine the mitochondrial membrane potential. Flow cytometry (j) and DCFH-DA (k) were used to detect ROS production in fluorescently labeled HK-2 cells. The data are presented as the mean ± SD of three independent experiments. ^∗^*P* < 0.05; ^∗∗^*P* < 0.01, versus the NC group, #*P* < 0.05, versus the COM+si-NC group, as determined by one-way ANOVA (a–d, f–k).

HK-2 cells were cultured with different doses of COM crystals for different durations to determine the optimal concentration and pretreatment time. We ultimately chose 100 *μ*g/ml as the treatment concentration and 8 h as the precondition duration (Figures 2(a) and 2(b)). To explore the effects of XIST, we transfected HK-2 cells with XIST siRNA and found that the RNA and protein levels of XIST, NLRP3, caspase-1, and IL-1*β* were significantly decreased (Figures 2(c)–2(f) and Supplementary Figure S2(a)). Then, XIST was knocked down, which clearly decreased the COM-induced LDH levels, H_2_O_2_ concentrations, and intercellular MDA generations in vitro; in contrast, the SOD levels were increased after XIST knockdown (Figure 2(g)). Flow cytometry analysis of PI staining showed that the amount of necrosis was decreased after transfection with si-XIST (Figure 2(h)). TMRE was used to further explore the mitochondrial injury and revealed that si-XIST alleviated the COM-induced loss of mitochondrial membrane potential (Figure 2(i)). Moreover, the ROS production was substantially decreased in the si-XIST group compared to the COM + si-NC group (Figures 2(j) and 2(k)). In summary, we found that suppression of XIST could prohibit the COM-induced inflammatory response and oxidative stress.

### 4.3. MiR-223-3p Directly Bound to the 3′-UTR of XIST to Suppress Its Expression

**Figure 3:**
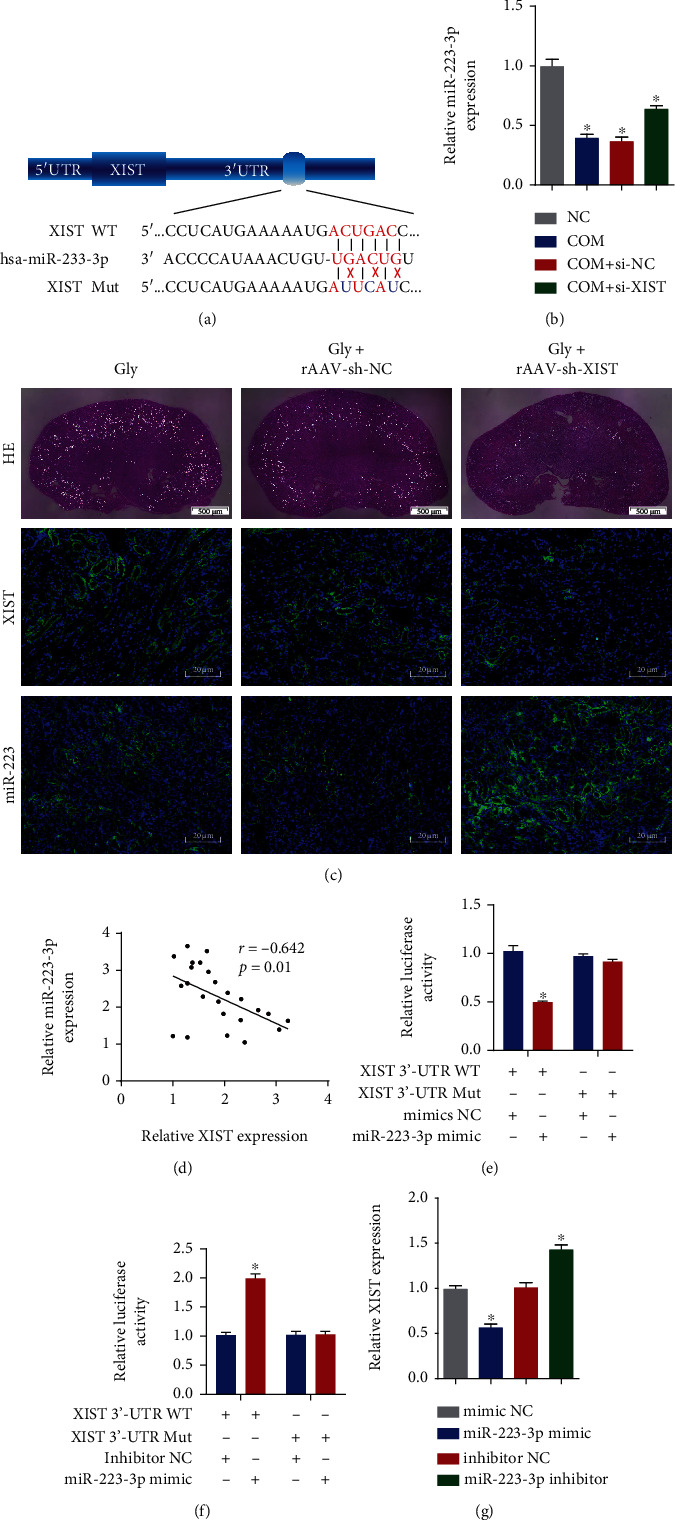
miR-223-3p directly bound to the 3′-UTR of XIST to suppress its expression. (a) Diagrammatic sketch of the WT and mutated XIST 3′-UTR targeting miR-223-3p. (b) qRT-PCR analysis was performed to determine the level of miR-223-3p in HK-2 cells. (c) Polarizing microscopy was performed to verify the crystal deposition in the CaOx nephrocalcinosis model. Fluorescence in situ hybridization was performed to examine the expression of XIST and miR-223-3p in the CaOx nephrocalcinosis model under different treatments. (d) The relationship between XIST and miR-223-3p was determined by Pearson analysis. The interaction of the 3′-UTR of XIST and the miR-223-3p mimics (e) or inhibitor (f) was verified via the dual-luciferase reporter gene assay. (g) The XIST levels in HK-2 cells were quantified by qRT-PCR. The data are presented as the mean ± SD of three independent experiments. ^∗^*P* < 0.05; ^∗∗^*P* < 0.01, as determined by one-way ANOVA (b, e–g) and Pearson's correlation test (d).

The interaction of lncRNAs and miRNAs is a core mechanism that exerts regulatory effects. To further explore the mechanism by which XIST affects CaOx nephrocalcinosis, we focused on miR-223-3p, which was identified as a potential binding target of XIST via StarBase 3.0, TargetScan and miRWalk (Figure 3(a)). We first detected the miR-223-3p level after siRNA transfection and found that its expression was enhanced compared to that in the si-NC group (Figure 3(b)). Meanwhile, FISH was performed to detect the expression levels of XIST and miRNA-223 in the renal tissues of CaOx nephrocalcinosis mice treated with rAAV-sh-XIST (Figure 3(c)), demonstrating that the levels of XIST were negatively correlated with the levels of miRNA-223 (Figure 3(d)). We constructed wild-type (WT) and mutant (Mut) XIST 3′-UTR luciferase reporter vectors to determine whether miR-223-3p mimics interact with XIST. The luciferase activity was notably inhibited in the WT group, while the activity in the Mut group was not significantly altered (Figure 3(e)). Transfection of the miR-223-3p inhibitor increased the luciferase activity in the WT group but did not significantly change that in the Mut group (Figure 3(f)). Additionally, the expression of XIST was decreased in HK-2 cells transfected with the miR-223-3p mimics and increased in those transfected with the miR-223-3p inhibitor (Figure 3(g)). These results suggested that the 3′-UTR of XIST directly binds to miR-223-3p and participates in biological regulation.

### 4.4. MiR-223-3p Inhibited the Expression of NLRP3 by Directly Binding to Its 3′-UTR

**Figure 4:**
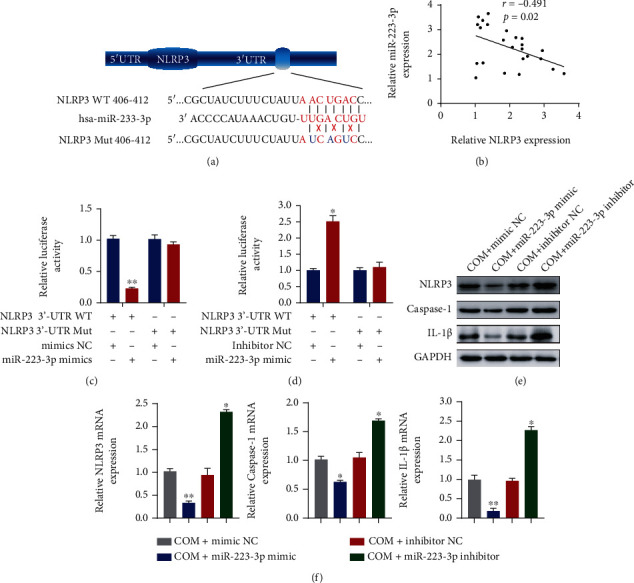
miR-223-3p inhibited the NLRP3 expression by directly binding to its 3′-UTR. (a) Diagrammatic sketch of the WT and mutated NLRP3 3′-UTR targeting miR-332-3p. (b) Pearson correlation analysis of NLRP3 and miR-223-3p was performed. A dual-luciferase reporter assay was performed to examine the interaction between the 3′-UTR of NLRP3 and the miR-223-3p mimics (c) or inhibitor (d). The expression levels of NLRP3, Caspase-1, and IL-1*β* in HK-2 cells transfected with the miR-223-3p mimics or inhibitor were detected by Western blotting (e) and qRT-PCR (f). The data are presented as the mean ± SD of three independent experiments. ^∗^*P* < 0.05; ^∗∗^*P* < 0.01, as determined by one-way ANOVA (c–f) and Pearson's correlation test (b).

We performed bioinformatics analysis to determine whether the 3′-UTR of NLRP3 contains a binding site for miR-223-3p (Figure 4(a)). We first found that the levels of NLRP3 were negatively associated with the levels of miR-223-3p (Figure 4(b)). To further verify the interaction, we constructed luciferase reporter vectors encoding the 3′-UTR of NLRP3 with the WT and Mut miR-223-3p binding sequences. After cotransfection with the miR-223-3p mimics, the luciferase activity in the WT group was substantially suppressed, while that in the Mut group was not substantially altered (Figure 4(c)). The miR-223-3p inhibitor was then transfected together with the two luciferase reporter vectors, and the luciferase activity in the WT group was notably elevated, while that in the Mut group was not substantially altered (Figure 4(d)). These experiments indicated that miR-223-3p directly binds to the 3′-UTR of NLRP3. To explore the function of miR-223-3p in CaOx-induced nephrocalcinosis and renal injury, we transfected miR-223-3p mimics and an inhibitor into COM-treated HK-2 cells. qRT-PCR and Western blot showed that miR-223-3p activation led to decreases in the expression of NLRP3, caspase-1, and IL-1*β*, while miR-223-3p inhibition caused the opposite phenomenon (Figures 4(e) and 4(f) and Supplementary Figure S2(b)). These data suggest that miR-223-3p inhibits NLRP3 expression by directly binding to its 3′-UTR.

### 4.5. MiR-223-3p Reversed the Effect of XIST on COM-Induced Renal Tubular Epithelial Cell Inflammation and Oxidative Injury

**Figure 5:**
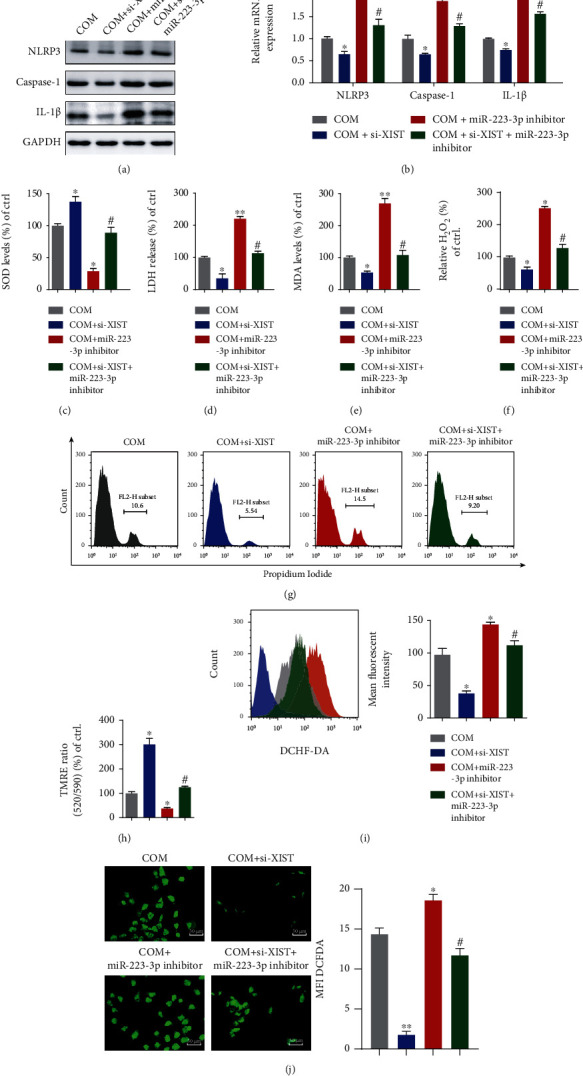
miR-223-3p reversed the effect of XIST on COM-induced renal tubular epithelial cell inflammation and oxidative injury. The levels of NLRP3, Caspase-1, and IL-1*β* in HK-2 cells cotransfected with si-XIST, the miR-223-3p inhibitor, or both were examined by Western blot (a) and qRT-PCR (b). The SOD expression (c), LDH release (d), MDA levels (e), and H_2_O_2_ generation (f) were detected in HK-2 cells transfected with si-XIST, the miR-223-3p inhibitor or si-XIST, and the miR-223-3p inhibitor in combination. (g) HK-2 necrosis was determined by flow cytometry. (h) The mitochondrial membrane potential was detected at Ex/Em wavelengths of 520/590 nm. Flow cytometry (i) and DCFH-DA (j) were used to assess the ROS production in HK-2 cells treated with si-XIST, the miR-223-3p inhibitor or si-XIST, and the miR-223-3p inhibitor in combination. The data are presented as the mean ± SD of three independent experiments. ^∗^*P* < 0.05; ^∗∗^*P* < 0.01, versus the COM group, #*P* < 0.05, versus the COM+inhibitor 223 group, as determined by one-way ANOVA (a–j).

To verify the internal functions of XIST and miRNA-223 in vitro, Western blot and qRT-PCR were performed, revealing that decreased levels of NLRP3, caspase-1, and IL-1*β* in COM-treated HK-2 cells cotransfected with si-XIST and the miRNA-223 inhibitor compared with those transfected with the miRNA-223 inhibitor alone (Figures 5(a) and 5(b) and Supplementary Figure S2(c)). Then, we detected the SOD levels after transfection with si-XIST, the miR-233 inhibitor, or both. The SOD expression was notably decreased by the miR-223-3p inhibitor, whereas si-XIST partially reversed this effect (Figure 5(c)). Additionally, the LDH levels, H_2_O_2_ concentrations, and intercellular MDA production showed opposite outcomes compared to the level of SOD (Figures 5(e)–5(f)). Moreover, PI staining was performed to detect COM-induced HK-2 necrosis (Figure 5(g)), demonstrating that si-XIST significantly reduced COM-induced HK-2 necrosis, while the miR-223-3p inhibitor abolished this treatment effect. In addition, si-XIST alleviated the COM-induced loss of mitochondrial membrane potential, while the combination of miR-223-3p inhibitor and si-XIST enhanced the COM-induced loss of mitochondrial membrane potential (Figure 5(h)). Moreover, the ROS burst was enhanced by the miR-223-3p inhibitor and attenuated by the miR-223-3p inhibitor and si-XIST in combination (Figures 5(i) and 5(j)). Altogether, these results suggest that the inhibition of miR-223-3p enhances the inflammatory response and ROS production, while si-XIST attenuates these effects and decreases the kidney tubular cell damage.

### 4.6. XIST Inhibition Alleviated CaOx Crystal Deposition and CaOx Nephrocalcinosis-Induced Inflammatory and Oxidative Kidney Injury via the XIST-miR-223-3p-NLRP3 ceRNA Pathway

**Figure 6:**
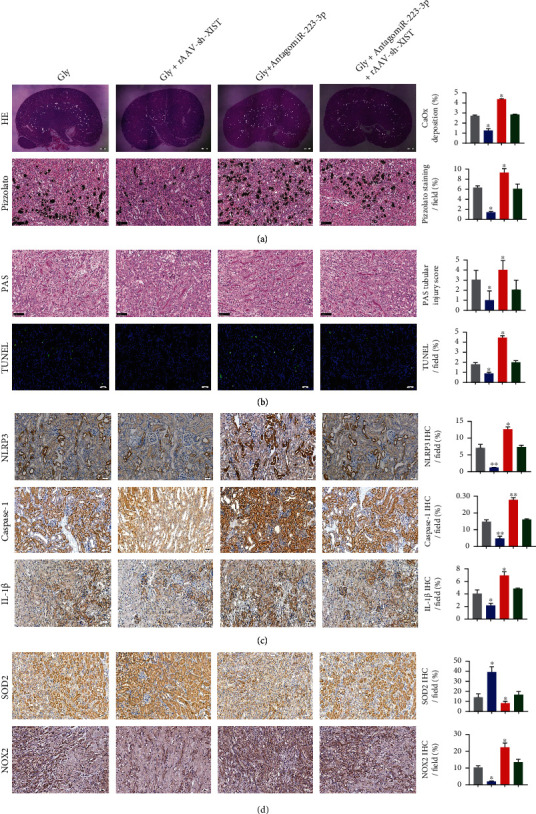
XIST inhibition alleviated CaOx crystal deposition and CaOx nephrocalcinosis-induced inflammatory and oxidative kidney injury via the XIST-miR-223-3p-NLRP3 ceRNA pathway. Polarization microscopy and Pizzolato staining were used to detect the crystal deposition in CaOx nephrocalcinosis model mice injected with rAAV-sh-XIST, antagomiR-223-3p, or both. (b) Kidney injuries were observed in CaOx nephrocalcinosis mice injected with rAAV-sh-XIST, antagomiR-223-3p, or both by PAS and TUNEL staining. The levels of NLRP3, Caspase-1, IL-1*β* (c), and SOD2 and NOX2 (d) in renal tissues were detected via IHC. The data are presented as the mean ± SD of three independent experiments. ^∗^*P* < 0.05; ^∗∗^*P* < 0.01, as determined by one-way ANOVA (a–d).

To verify the ability of the interaction between XIST and miR-223-3p in decreasing the CaOx deposition by suppressing renal inflammatory and oxidative injury in vivo, we administered the effects of rAAV-sh-XIST, antagomiR-223-3p, or both to CaOx nephrocalcinosis mice. Next, HE and Pizzolato staining was performed, and the cells were observed under the polarized optical microscope. The results showed that silencing XIST notably decreased the CaOx deposition, while inhibiting miR-223-3p significantly increased the CaOx deposition (Figure 6(a)). Moreover, PAS and TUNEL staining suggested that the kidney injury was less extensive in the rAAV-sh-XIST group and more extensive in the antagomiR-223-3p group (Figure 6(b)). On the other hand, the IHC results indicated that the expression levels of the proinflammatory markers NLRP3, Caspase-1, and IL-1*β* and the prooxidant NOX2 were prominently decreased after rAAV-sh-XIST injection and significantly increased after antagomiR-223-3p administration, while the SOD2 expression showed the opposite changes (Figures 6(c) and 6(d)). In addition, no significant changes were observed in CaOx nephrocalcinosis mice injected with both rAAV-sh-XIST and antagomiR-223-3p. These results indicate that miR-223-3p reversed the effect of XIST on COM crystal-induced renal tubular epithelial cell inflammation and oxidative stress injury (Figures 6(a)–6(d)). Therefore, downregulation of XIST can attenuate the ROS burst, protect renal tubular cell against injury, and further decrease crystal deposition in subjects with CaOx nephrocalcinosis.

**Figure 7:**
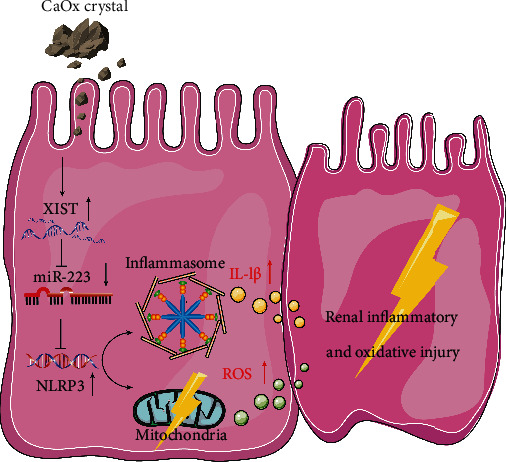
Schematic diagram XIST inhibition decreases CaOx crystal-induced inflammatory and oxidative kidney injury by interacting with miR-223-3p and the NLRP3/Caspase-1/IL-1*β* pathway.

Taken together, these results indicate that XIST inhibition decreases CaOx crystal-induced inflammatory and oxidative kidney injury by interacting with miR-223-3p and the NLRP3/Caspase-1/IL-1*β* pathway (Figure 7).

## 5. Discussion

Kidney stone formation is ascribed to multiple factors, including infection, metabolism, and other diseases [1, 3]. The progression of kidney stone formation involves CaOx crystal adhesion, growth, and advancement in renal tubules and the pelvis. Previous studies revealed that renal tubular epithelial cells exposed to CaOx generated excess ROS and an inflammatory response, thereby leading to further renal injury and increased CaOx deposition [21–23]. Therefore, the development of diagnostic markers and targeted treatments that delay or attenuate the progression of CaOx nephrocalcinosis is highly essential.

An increasing number of studies have indicated that lncRNAs play irreplaceable roles in multiple transcriptional and posttranscriptional biological processes, including chromatin modification, DNA repair, preservation of mRNA stability, and miRNA sponging [24–26]. XIST was one of the first lncRNAs to be discovered in the 1990s and participates in the regulation of many cancer-related processes and the inflammatory response [14, 27]. XIST, which also acts as a molecular sponge for miR-133a-3p, was shown to act as a ceRNA to reverse the repression of RohA and promote the progression of inflammatory colorectal cancer [28]. Additionally, XIST enhanced lipopolysaccharide- (LPS-) induced acute respiratory distress syndrome by upregulating the levels of interferon regulatory factor 2 by binding to miR-204 [29]. Hence, we hypothesized that the lncRNA XIST is a crucial factor that participates in the pathophysiological processes of inflammatory diseases. In this study, we first unveiled the ceRNA mechanism by which XIST regulates CaOx-induced inflammation and ROS disorders.

Activation of the NLRP3 inflammasome leads to the initial stages and progression of many diseases, including kidney stones [30, 31]. NLRP3 knockdown sufficiently ameliorated podocyte autophagy and protected podocytes from diabetic nephropathy-induced injury [32]. Moreover, Ying et al. demonstrated that NLRP3 interacted with miR-495, whose increased expression inhibited the activation of the NLRP3 inflammasome and downregulated inflammatory cell infiltration and responses in subjects with acute lung injury [33]. Additionally, NLRP3 activation contributed to robust ROS production and inflammatory responses via TXNIP in subjects with hyperoxaluria and in a kidney CaOx rat model [34]. In accordance with these findings, our experiments suggested that NLRP3 was activated in the CaOx-induced renal tubular epithelial cell injury in vitro and in vivo model.

NLRP3 was shown to be activated by the crystal deposition of monosodium urate into the joints, which induced inflammatory responses, and another study found that NLRP3-deficient mice were protected from crystal-related injuries [35, 36]. Moreover, NLRP3 blockade was shown to alleviate the inflammatory injury induced by cholesterol crystals in atherosclerosis [37, 38]. Anders et al. found that NLRP3-deficient mice fed an oxalate-rich diet failed to develop CaOx nephrocalcinosis and that their kidneys were protected from nephrocalcinosis-related fibrosis [39]. In addition, Mulay et al. showed that intrarenal calcium oxalate crystal deposition caused tubular damage, inflammation, and renal failure via the dendritic cell (DC) secretion of inflammatory cytokines and that IL-1*β* blockade decreased tubular injury [31]. Overall, NLRP3 is closely related to diseases induced by different kinds of crystals, including CaOx crystals.

To explore the internal relationship between the changes in XIST and NLRP3, we focused on the ceRNA hypothesis. We found that miR-223-3p may be a specific bridge between XIST and NLRP3, but the role of miR-223-3p in CaOx nephrocalcinosis has not been described. MiRNAs alter the gene expression by targeting mRNAs in the posttranscriptional regulation period [40]. MiR-223-3p is considered a key regulator of inflammation and immune processes, ranging from myeloid differentiation to neutrophil function, macrophage polarization, and activation [41–43]. Neudecker et al. indicated that the expression of miR-223-3p was increased in subjects with inflammatory bowel disease (IBD) and that it limited the inflammatory response by inhibiting the NLRP3 inflammasome in the intestinal nonspecific immune responses [44]. Consistent with previous studies, we discovered that miR-223-3p bound to the 3′-UTRs of both the XIST and NLRP3 and was associated with their cooperation with each other. In addition, the elevated expression of miR-223-3p significantly inhibited the mRNA translated products of NLRP3.

Taken together, these results showed that XIST enhanced the translation of NLRP3 by competitively binding to miR-223-3p to thereby release NLRP3 mRNA; these phenomena exacerbated the inflammatory response and ROS production, leading to renal tubular epithelial cell injury. This new mechanistic insight into the molecular regulatory function of lncRNAs provided by our research will be crucial for the further exploration of novel diagnostic and therapeutic strategies for nephroliths.

## Data Availability

The data used to support the findings of this study are included within the article.

## Supplementary Materials

Supplementary Materials Supplementary Table 1: list of primer sequences used for the real-time qPCR analysis. Supplementary Figure S1: immunofluorescence analysis of GFP and XIST expression in mouse kidneys. (A) Mice were injected with rAAV vector or rAAV-sh-XIST (rAAV-2/9-eGFP) via the tail vein. At eight-week postinjection, their kidneys were snap frozen and whole mounted for imaging (magnification, ×40). (B) qPCR analysis of XIST in mouse kidneys. GAPDH was used as the internal control. The data are shown as the mean ± SD. One representative plot of *n* = 6 samples is shown. ^∗^*P* < 0.05; ^∗∗^*P* < 0.01, as determined by Student's *t*-test. Supplementary Figure S2: relative quantification of the protein expression of NLRP3, Caspase-1, and IL-1*β* by in Western blot. GAPDH was used as the internal control. The data are shown as the mean ± SD. ^∗^*P* < 0.05; ^∗∗^*P* < 0.01, as determined by one-way ANOVA (A–D).
