# Supplementary material for: XIST Inhibition Attenuates Calcium Oxalate Nephrocalcinosis-Induced Renal Inflammation and Oxidative Injury via the miR-223/NLRP3 Pathway
Source: Oxid Med Cell Longev. 2021 Sep 2;2021:1676152. doi: 10.1155/2021/1676152 (PMC8429007; doi:10.1155/2021/1676152)
Supplement: Supplementary Materials — Supplementary Table 1: list of primer sequences used for the real-time qPCR analysis. Supplementary Figure S1: immunofluorescence analysis of GFP and XIST expression in mouse kidneys. (A) Mice were injected with rAAV vector or rAAV-sh-XIST (rAAV-2/9-eGFP) via the tail vein. At eight-week postinjection, their kidneys were snap frozen and whole mounted for imaging (magnification, ×40). (B) qPCR analysis of XIST in mouse kidneys. GAPDH was used as the internal control. The data are shown as the mean ± SD. One representative plot of n = 6 samples is shown. ∗P < 0.05; ∗∗P < 0.01, as determined by Student's t-test. Supplementary Figure S2: relative quantification of the protein expression of NLRP3, Caspase-1, and IL-1β by in Western blot. GAPDH was used as the internal control. The data are shown as the mean ± SD. ∗P < 0.05; ∗∗P < 0.01, as determined by one-way ANOVA (A–D). [file 1676152.f1.docx]

**Supplementary Materials**

**Supplementary Table 1.** List of primer sequences used for the real-time qPCR analysis.

| **Species** | **Name** | **(5'-3')** | **Sequence (5'-3')** |
| --- | --- | --- | --- |
| Human | XIST | Forward | ATGGACAGTGCTGGATTG |
|  |  | Reverse | CAGGTCTTCGCTGAGTAG |
|  | NLRP3 | Forward | GATCTTCGCTGCGATCAACAG |
|  |  | Reverse | CGTGCATTATCTGAACCCCAC |
|  | Caspase-1 | Forward | TTTCCGCAAGGTTCGATTTTCA |
|  |  | Reverse | GGCATCTGCGCTCTACCATC |
|  | IL-1β | Forward | ATGATGGCTTATTACAGTGGCAA |
|  |  | Reverse | GTCGGAGATTCGTAGCTGGA |
|  | GAPDH | Forward | ACAACTTTGGTATCGTGGAAGG |
|  |  | Reverse | GCCATCACGCCACAGTTTC |
|  | hsa-miR-223-3P | Forward | GCGCGTGTCAGTTTGTCAAAT |
|  |  | Reverse | AGTGCAGGGTCCGAGGTATT |
|  | U6 | Forward | CTCGCTTCGGCAGCACA |
|  |  | Reverse | AACGCTTCACGAATTTGCGT |
| Mouse | XIST | Forward | TAGTCCTCTGCGGCTTCC |
|  |  | Reverse | TGCTGATCGTTTGGTGCT |
|  | NLRP3 | Forward | TGTGAGAAGCAGGTTCTACTCT |
|  |  | Reverse | TGTAGCGACTGTTGAGGTCCA |
|  | Caspase-1 | Forward | ACAAGGCACGGGACCTATG |
|  |  | Reverse | TCCCAGTCAGTCCTGGAAATG |
|  | IL-1β | Forward | GAAATGCCACCTTTTGACAGTG |
|  |  | Reverse | TGGATGCTCTCATCAGGACAG |
|  | GAPDH | Forward | AGGTCGGTGTGAACGGATTTG |
|  |  | Reverse | TGTAGACCATGTAGTTGAGGTCA |
|  | mmu-miR-223-3P | Forward | GCGCGTGTCAGTTTGTCAAAT |
|  |  | Reverse | AGTGCAGGGTCCGAGGTATT |
|  | U6 | Forward | CTCGCTTCGGCAGCACA |
|  |  | Reverse | AACGCTTCACGAATTTGCGT |

**
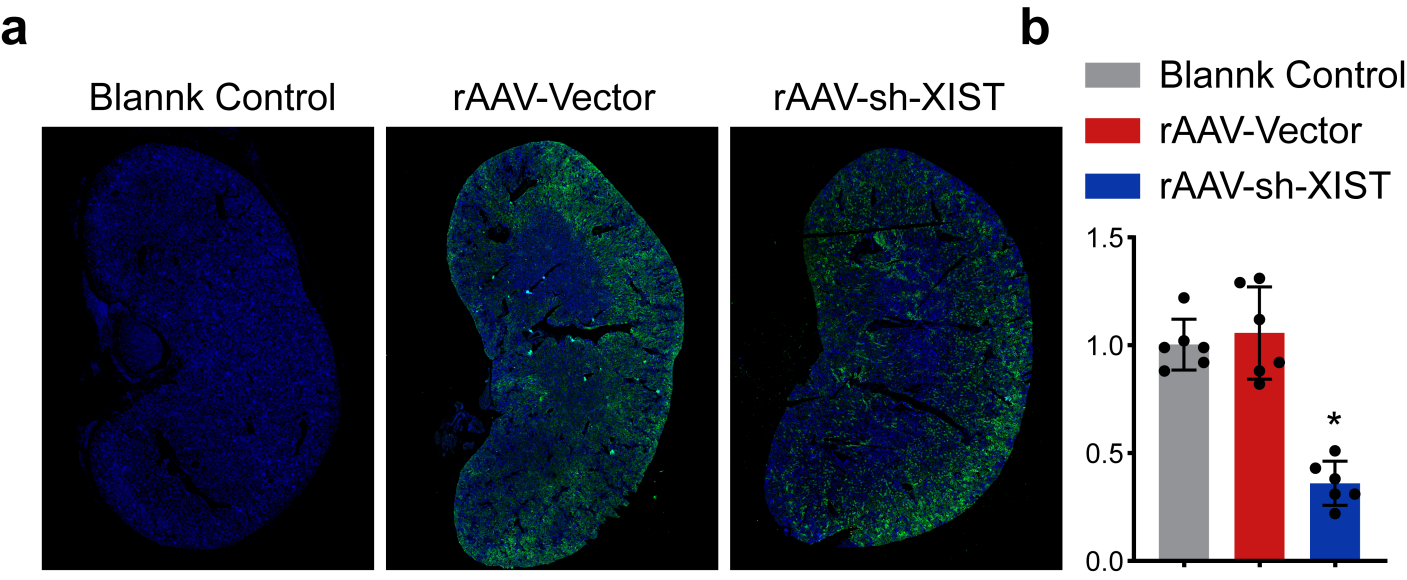
**

**Supplementary Figure S1. [Immunofluorescence](https://www.sciencedirect.com/topics/biochemistry-genetics-and-molecular-biology/immunofluorescence) analysis of GFP and XIST expression in [mouse](https://www.sciencedirect.com/topics/biochemistry-genetics-and-molecular-biology/mouse) kidneys.**

(a) Mice were injected with rAAV vector or rAAV-sh-XIST (rAAV-2/9-eGFP) via the tail vein. At eight weeks postinjection, their kidneys were snap frozen and whole mounted for imaging (magnification, ×40). (b) qPCR analysis of XIST in mouse kidneys. GAPDH was used as the internal control. The data are shown as the mean±SD. One representative plot of n = 6 samples is shown. *P < 0.05; **P < 0.01, as determined by Student’s t-test.

**
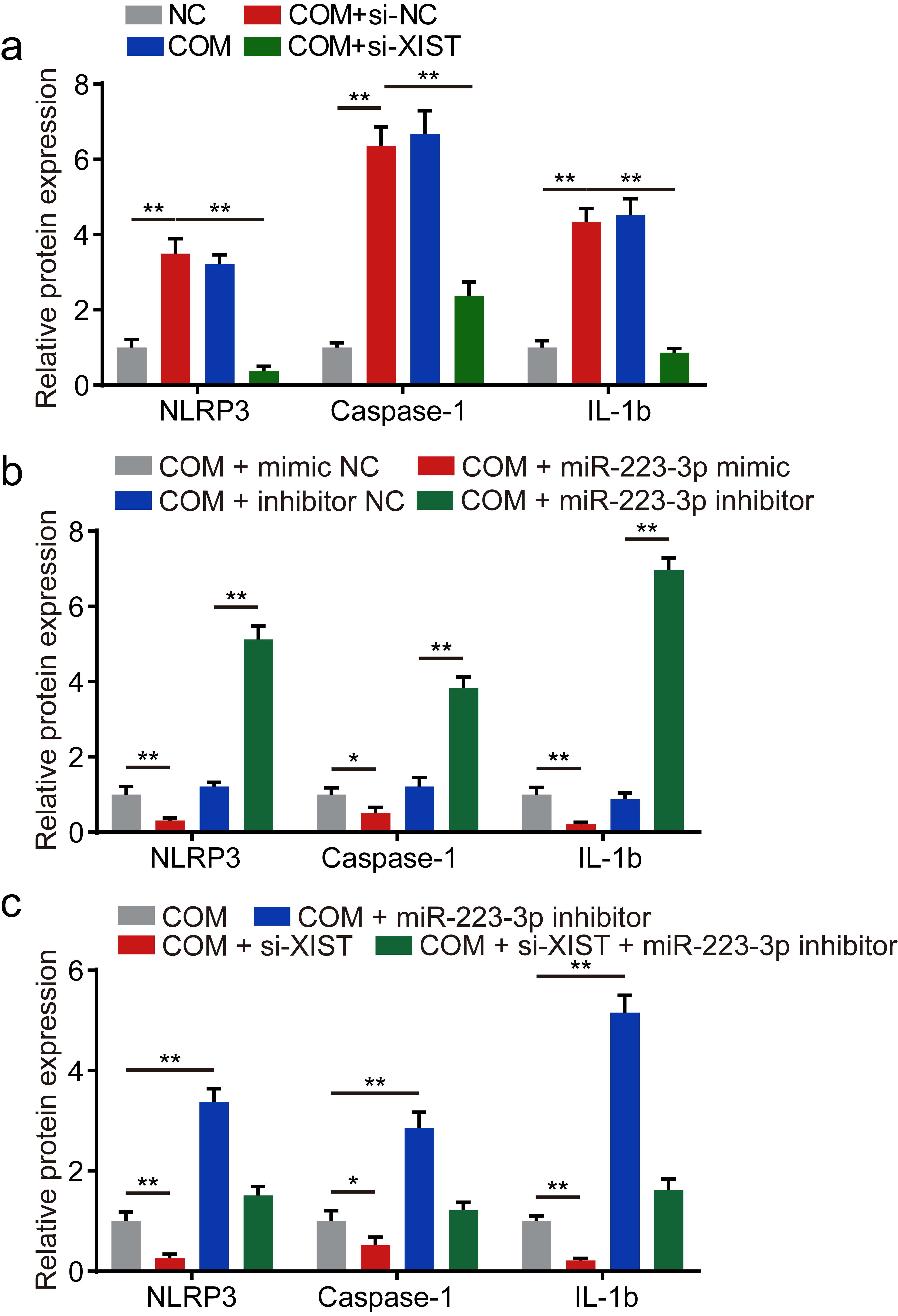
**

**Supplementary Figure S2.** Relative quantification of the protein expression of NLRP3, Caspase-1 and IL-1β by in Western blot. GAPDH was used as the internal control. The data are shown as the mean±SD. *P < 0.05; **P < 0.01, as determined by one-way ANOVA (a-d).
